# Virtual reality perceptual training can improve the temporal discrimination ability of swinging during softball batting

**DOI:** 10.3389/fspor.2024.1332149

**Published:** 2024-02-21

**Authors:** Daiki Nasu, Takamichi Baba, Takumi Imamura, Masumi Yamaguchi, Yoshitake Kitanishi, Makio Kashino

**Affiliations:** ^1^ NTT Communication Science Laboratories, NTT Corporation, Kanagawa, Japan; ^2^ Biostatistics Center, Shionogi & Co, Ltd., Osaka, Japan; ^3^ Department of Data Science, Shionogi & Co, Ltd., Osaka, Japan

**Keywords:** perception-action coupling, temporal occlusion paradigm, two-visual-pathway hypothesis, head-mounted display (HMD), fastball sports, hitting motion

## Abstract

Perception and action uncoupling in fastball sports anticipatory training is often criticized. Nevertheless, perception-only training offers distinct advantages, such as flexibility concerning time, place, and injury limitations. Therefore, the effectiveness of this training approach warrants evaluation. This study developed a virtual reality (VR) training system based on the idea that the two visual pathways in the brain are associated with visual information attributes, rather than perception or action. A key feature of this study's perception-only training was its presentation of not only the opponent's kinematics but also the ball's flight information (the attributes that guide hitting) to train the visual system necessary for real situations. Seventeen female softball batters were assigned to two groups: a training group (*N* = 9) and a control group (*N* = 8). Only the training group underwent the VR anticipatory skill training to discriminate the different ball speeds. Both groups completed a perception test and an action test on the VR during the pre- and post-training periods. The perception test assessed response accuracy (RA) in discriminating ball speed, and the action test assessed the temporal difference in swing onset (delta onset). Comparison of these two outcome variables between groups revealed that perception-only training improved both perceptual and action responses. This study demonstrated the effectiveness of perception-only training and emphasized the need for its thoughtful utilization and design.

## 1 Introduction

In fastball sports such as baseball, softball, cricket, and tennis, it is extremely important to anticipate the speed, location, and type of ball thrown by an opponent, based on the opponent's kinematics and ball flight information. Such anticipatory skills of expert athletes are superior to those of non-experts (1, 2), and the experimentally assessed anticipatory skills of athletes are related to their actual game performance (3–5). Many sports scientists, coaches, and athletes are interested in whether these skills can be effectively enhanced through additional training.

The temporal occlusion paradigm is the most common method used for assessing and training anticipatory skills (6–8). Typical training studies with this paradigm involve anticipating the location and/or type of ball by observing video footage of the opponent's motion and ball, occluded at several points in time (3). This allows players to anticipate the fate of the ball from as little information as possible, at the earliest possible time. Traditionally, observers respond in a manner that reflects their perceptual skills, either verbally, in writing, or by pressing a button. However, assessing and training anticipatory skills in this manner received some criticism, since it lacks the means required in actual sports actions (e.g., bat swings) (9, 10). They argue that to correctly assess sports performance and maximize the effects of training, the same should be conducted in an environment that replicates the “in-situ” conditions as much as possible. However, their argument is just a perspective and there are no empirical studies that clearly demonstrate that perceptual skills can be assessed/trained only when coupled with sports actions (11).

One of the grounds for such claim is the two-visual-pathway hypothesis, which states that there are two independent visual processing pathways in the brain: the ventral and dorsal pathways (12–14). According to this hypothesis, the “vision for perception” is primarily processed in the ventral pathway and provides perception information regarding object features and their relationships for identification and classification. Conversely, “vision for action” is primarily processed in the dorsal pathway and mediates visual control of selected actions. Therefore, the pathway for anticipating the ball's fate (ventral) and the pathway for hitting the ball (dorsal) differ, and there are various discussions regarding the effectiveness of training only the former (15, 16).

However, there are some objections to the two-visual-pathway hypothesis. Some have argued that the two visual pathways are not associated with perception and action but with attributes of visual information. The ventral pathway has higher spatial resolution and is associated with visual information related to object recognition, such as color and shape, whereas the dorsal pathway has higher temporal resolution and is associated with information that guides action, such as the speed and direction of object motion (17–19). De la Malla and colleagues found that if an attribute of visual information is a guiding action, such as the speed of an object, it is processed in the dorsal pathway regardless of whether one perceives the object or interacts with it (20, 21). Moreover, behavioral and neurophysiological studies have evidenced that the two pathways are interconnected, and their dissociation was recently dismissed (19, 22). The theory of the two visual pathways, their connection to perception and action, and their dissociation have recently been revisited, and Goodale and Milner discussed revisions to their original theory (23, 24).

In anticipatory skill training, it is essential to consider the characteristics of visual processing and to create protocols for real sports situations. The superiority of experts in fastball sports over non-experts lies in their ability to acquire useful perceptual information for hitting a ball from probabilities such as the kinematics of the opponent's movement and the early flight of the ball (1). That is, the focus should be on training the visual system with action-guiding attributes rather than the specific physical action. Perceptual training, even without action, can then potentially improve motor performance. Perception-only training can be conducted at any time and place, which significantly benefits individual training or athletes whose physical activity is limited due to injury. Consequently, it is valuable to verify and discuss its effectiveness. This study developed a perception-only training protocol for softball batters to examine whether the anticipatory skills training intervention could improve their motor performance. The training protocol was characterized by three features: (1) focusing on the ball speed discrimination ability, (2) adopting the progressive temporal occlusion paradigm, and (3) training and testing in a virtual reality environment using a head-mounted display (HMD-VR).

First, this study aimed to train the batter's anticipatory skill regarding pitch types at different speeds (fastball and change-up) and examined the intervention effects on action by observing the temporal difference of swing onset (delta onset) when the batter faced the two types of balls. The speed of an object is considered an attribute processed by the dorsal stream (20, 21). One study also found that ball speed discrimination in softball batting is processed similarly to perception and action (25). Thus, this study focused on improving batters' ability to anticipate ball speed through perceptual training, rather than spatial location anticipation. Since this study aimed to train anticipatory skills, the focus was on swing onset, where anticipatory skills are directly reflected, rather than on impact time, which includes the ability to adjust the swing after movement initiation. The delta onset is an essential factor for softball batters and is related to their performance during an actual game (5).

Second, a progressive occlusion paradigm was adopted during training, in which the occlusion timing of the ball trajectory was progressively preponed depending on the training step. In ball sports, the available sources of information for anticipating the ball's fate include ball flight and opponent kinematics. The ball is not only an object to be observed, but an object to be interacted with. Thus, ball flight information guides action and might be dominated by the dorsal pathway, regardless of the presence of action (20, 21). Additionally, the biological motion (i.e., opponent's kinematics) includes both “form” and “motion” and its processing can involve both ventral and dorsal pathways (22). In the absence of ball flight information, without action, the prediction accuracy of the throw direction in cricket was at the chance level, diminishing skilled players' advantage (26). The verbal prediction based only on the opponent's kinematics may have led participants to focus on the “form” discrimination and to use a different system than the one trained by experts. Therefore, a series of information, including pitching kinematics and ball flight, should be presented. However, predicting ball speed utilizing the ball flight information presented throughout training is too easy to be effective in training. To differentiate the swing onset, the ball speed should be accurately identified at the earliest possible time. Consequently, this study adopted the progressive occlusion paradigm. In particular, ball flight information is not eliminated until the final stage of training.

Third, HMD-VR was used for perceptual training and testing. VR provided depth information, particularly concerning ball trajectory, and was expected to have a greater training effect than 2D displays. Previous studies reported that the timing control of swinging motion was more accurate with HMD-VR than with the 2D display in baseball batting (27) and that HMD-VR is a valid method of evaluating the delta onset in softball batting (28). Moreover, the use of HMDs has been recommended as a way to compensate for the insufficiency of 2D displays (29). The perceptual training and pre- and post-tests were all conducted in the VR environment, and the transfer test to the real environment was not included in this study.

## 2 Materials and methods

### 2.1 Participants

Seventeen elite female softball batters voluntarily participated in this study. Participants were competitive fast-pitch softball players from the Japan Softball Top League. This study only recruited participants from the same team due to restrictions on the use of pitchers’ images in the VR and training period constraints. Consequently, the sample size was not based on statistical evidence; however, as many participants as possible participated. Seventeen batters were randomly assigned to one of two groups, the training group (with perceptual training; *N* = 9) or the control group (without perceptual training; *N* = 8) (Table 1). All participants usually trained approximately six hours a day, six times a week, including general batting training, but did not partake in any 2D video or VR training. Participants provided written informed consent before participating in the experiments. This study was approved by the Shionogi Research Ethics Committee (Osaka, Japan; the approval code EP20-18) and was in accordance with the Declaration of Helsinki.

**Table 1:** Participants.

	Training (*n* = 9)	Control (*n* = 8)
Age (yrs) [Mean ± SD]	24.9 ± 2.9	23.5 ± 4.0
Height (m) [Mean ± SD]	1.64 ± 0.05	1.61 ± 0.04
Softball and baseball career (yrs) [Mean ± SD]	14.7 ± 3.5	16.6 ± 4.1
Handedness	4 right- and 5 left-handed batters.	3 right- and 5 left-handed batters.

### 2.2 VR system

This study used a VR system with a wireless HMD (Meta Quest 2; Meta Platforms Inc., Menlo Park, CA, USA) for all pitch speed anticipation-related tests and training. This VR system was the same as that used in a previous study and was valid for the assessment of pitch speed anticipation ability with action (28).

**Figure 1:**
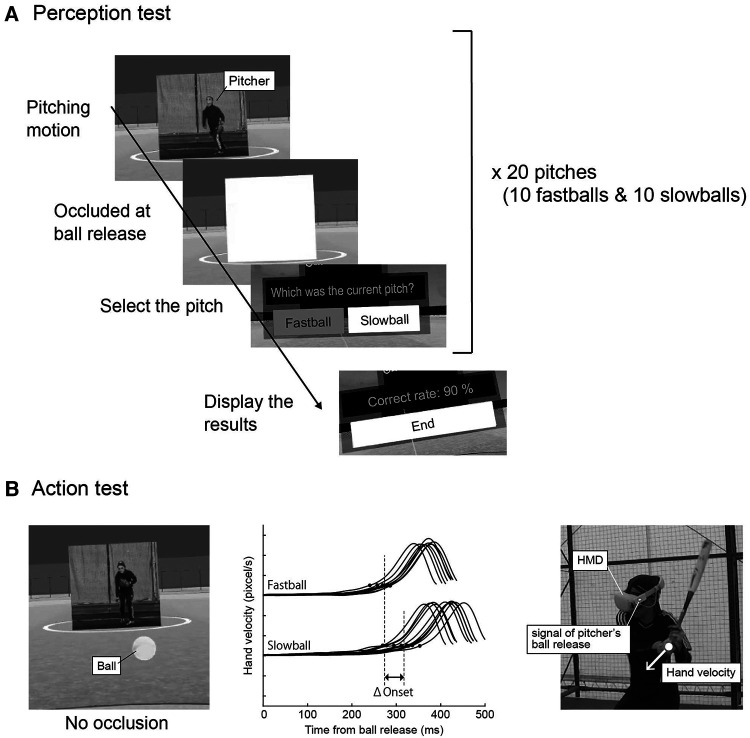
Perception and action test in pre- and post- training. (**A**) In the perception test, participants observed pitchers throwing ten fastballs and ten slowballs and selected the pitch type in the VR system. The images and balls were occluded at the time of the pitcher's ball release. The RA was presented after 20 pitches were completed. (**B**) In the action test, participants swung at ten fastballs and ten slowballs (no occlusion). The center panel represents time curves of hand velocity for a typical batter which were analyzed using video analysis, where the small dots indicate the swing onset and vertical dot line indicates their median values for each pitch type. The delta onset was calculated as the difference in the median values of swing onset between pitch types.

Two female pitchers (pitcher A, right-handed; pitcher B, left-handed) from the same team as the participating batters cooperated in the VR system construction. The pitching data for the two pitchers were obtained to render the pitchers and ball trajectories in VR. Each pitcher threw 30 fastballs and 30 slowballs (i.e., change-ups) to various locations in the strike zone, and their pitching motions were recorded by placing a video camera (60 fps; 1,920 × 1,080 px; Sports Coaching Cam, JVCKENWOOD Corp., Yokohama, Japan) behind the catcher. Additionally, the ball trajectory parameters were measured using Rapsodo 2.0 (Rapsodo LLC, Yokohama, Japan), which resulted in a total of 60 datasets per pitcher. 2D video images of the pitcher were rendered at the position of the pitcher rubbers within the VR softball field, and balls appearing in the video were removed to avoid duplicate representations (Figure 1), based on a previous study (27). Additionally, the 3D ball trajectories were rendered by approximating them as a quadratic time function based on the ball trajectories obtained by Rapsodo 2.0. The mean and SD of the ball speeds of pitcher A were 95.1 ± 0.9 km/h for the fastball and 68.5 ± 2.0 km/h for the slowball, and those of pitcher B were 92.5 ± 1.4 km/h and 73.4 ± 1.7 km/h, respectively. Since the VR system did not track the movement of the batter and bat, it did not allow interaction with objects in the virtual world.

### 2.3 Pre and post test

This study aimed to determine whether a perceptual training intervention would improve swinging action: the temporal discrimination ability of swing onset at two pitch speeds. Therefore, both perception and action at the two pitch speeds were assessed pre- and post-training. Each participant faced one of the two pitchers, who was common to all the pre- and post-perception and action tests. The pairs of pitcher videos and 3D ball trajectories were randomly selected from the 60 datasets (30 for each pitch type) for each pitcher. The order of the perception and action tests at each test period was counterbalanced across participants.

#### 2.3.1 Perception test

The participants wore the HMD and stood in the batter's box in the VR environment with an HMD controller. First, as practice, they observed two fastballs and two slowballs in a situation with a known pitch type. Subsequently, they observed ten fastballs and ten slowballs randomly thrown by the pitcher in a situation with an unknown pitch type. They assumed a batting stance but did not swing. In both the pre- and post-tests, the ball trajectory and pitcher image were occluded at the moment of the pitcher's ball release. This meant that the participants had to identify the pitch type only through opponent kinematics. After each pitch was thrown, two candidate pitches were presented in the VR space (Figure 1A), and participants were asked to respond as quickly as possible by selecting one using the controller. After 20 pitches were completed, the percentage of correct responses to those pitches was calculated as response accuracy (RA) and presented to the participant. The RA values were transferred from the HMD to a computer after the test.

#### 2.3.2 Action test

Similar to the perception test, participants stood in the batter's box in the VR environment and faced the pitcher. However, they were instructed to hold the bat and swing toward the thrown balls. Following practice with two fastballs and two slowballs, they swung toward ten fastballs and ten slowballs, with an unknown pitch type. In contrast to the perception test, the pitcher's image and the ball trajectory were not occluded. Participants could neither hit the virtual ball in the system nor receive visual or tactile feedback. A video camera (240 fps; 640 × 480 px; Sports Coaching Cam, JVCKENWOOD Corp., Yokohama, Japan) was placed 5 m away from the participant to capture an LED signal of the timing of the pitcher's ball release and the batter's motion (Figure 1B).

### 2.4 Training protocol

Participants in the training group performed at least eight perceptual training sessions over four weeks. Participants in the control group, on the other hand, received no training intervention. Each training session was scheduled after the end of regular daily training and lasted approximately 15 min. The perceptual training procedure was identical to that of the perceptual test. However, in the training, two blocks of 20 pitches (10 of each pitch type) were performed in one session with a 5-minute break between the blocks, with a total of 40 pitches per session.

Additionally, the timing of occluding the pitcher's image and the ball trajectory was gradually preponed with each completed session; in the first session, all ball trajectories and pitcher images were presented (no occlusion; approximately 400 ms for fastball and approximately 550 ms for slowball); in the second session, the images were occluded at 300 ms after the pitcher's ball release, adapted by 50 ms thereafter (250 ms, 200 ms, 150 ms, 100 ms, 50 ms, 0 ms). Thus, in the early stage, the training was designed to strengthen the ability to anticipate ball speed from a series of information, including the pitching motion and ball trajectory, and to gradually improve anticipation ability. This gradual increase in difficulty also maintained participants' motivation. Participants who did not exceed 80% of the correct responses in each session were provided additional training with the same occlusion timing. Four participants exceeded 80% correct responses in all sessions, while five participants did not only in the 0 ms occlusion session.

### 2.5 Data analysis

Temporal discrimination ability was assessed by differentiating the time of swing onset for the two types of pitches at different ball speeds (delta onset) in pre- and post-action tests. The recorded videos of the participants' motions were analyzed using DeepLabCut (ver. 2.1.10.4), which is open-source software for markerless pose estimation based on transfer learning with deep neural networks (30, 31). A total of 60 frames × three trials per participant were selected and manually labeled the bottom hand (Figure 1B). Subsequently, the network was trained for 100,000 iterations until the loss plateaued and was used for automatic labeling. The 2D positions of the obtained markers were smoothened using a zero-lag fourth-order Butterworth low-pass filter with a cutoff frequency of 10 Hz. Since this study focused on the temporal aspects, it was unnecessary to calibrate for actual length conversion.

The swing onset was defined as the moment when the resultant hand velocity exceeded a certain threshold, which was 20% of the mean peak velocity for each batter (476.3 ± 74.9 pixel/s; Figure 1B). For each pitch type, trials that exceeded twice the median absolute deviation were excluded as outliers. The number of trials excluded as outliers across participants was 1.1 ± 1.2. The time of swing onset was defined as the duration between the pitcher's ball release (LED signal) and the swing onset and the median value for each pitch type was calculated. Subsequently, the delta onset was calculated as the difference in swing onset time between pitch types.

### 2.6 Statistics

SAS Version 9.4 (SAS Institute, Cary, NC, USA) was used for statistical analyses. This study aimed to determine the effects of a perceptual training intervention on two outcome variables (RA and delta onset). The RA values before the intervention were different between the two groups. In addition, each participant faced one of the two pitchers. Therefore, an Analysis of Covariance (ANCOVA) was conducted with these outcome variables as dependent variables, and the pitcher and pre-intervention values as covariates to determine the intervention effect group differences. The intervention effects (Y) was defined as the log-transformed ratio of pre- and post-training outcomes, which was assumed to follow a normal distribution.

(1) $$\begin{array}{c} Y=\log\left(\frac{y_{\mathrm{Post}}}{y_{\mathrm{Pre}}}\right) \end{array}$$

where *y* is the outcome, and Pre and Post refer to pre- and post-training, respectively. It is generally inappropriate to assume a normal distribution for the ratio of outcomes pre- and post-training because their range has a positive bias (0, ∞). However, the assumption is valid through log transformation because the range of log-transformed values is whole numbers (−∞, ∞). In the ANCOVA, *Y* was the response variable, group was the fixed effect, and log-transformed baseline values of the outcome and pitcher faced in the tests were used as covariates. The adjusted parameter of the group difference by ANCOVA is described as ${\bar{Y}}^{T}-{\bar{Y}}^{C}$, where the overlines indicate the means on the logarithm scale, and *T* and *C* superscripts represent the training and control groups, respectively. For interpretation, we exponentialized (back to antilogarithm) and subsequently transformed it as follows:

(2) $$\begin{array}{c} \frac{e^{\bar{Y^{T}}}}{e^{\bar{Y^{C}}}}\times 100 \end{array}$$

This parameter represents the between-group ratio of the geometric mean of the outcome; and a ratio exceeding 100% can be interpreted as the training group improving, compared to the control group. This parameter was calculated for both RA and delta onset and used as a group differences measure.

Hypothetical testing was not performed because the sample size was not planned to be statistically significant. The point estimate, its 95% confidence interval (CI), and the effect size of the group differences (Cohen's *d*) were calculated and the results were discussed based on the magnitude of the intervention effect (see the Discussion). The Cohen's *d* was calculated as the value of adjusted mean difference of *Y* between groups divided by pooled standard deviation of the *Y* for two independent samples. In addition, the correlation coefficient (Pearson's *r*) between RA and delta onset was calculated by pre- and post-training to investigate the relationship between them.

## 3 Results

### 3.1 Response accuracy (RA)

**Figure 2:**
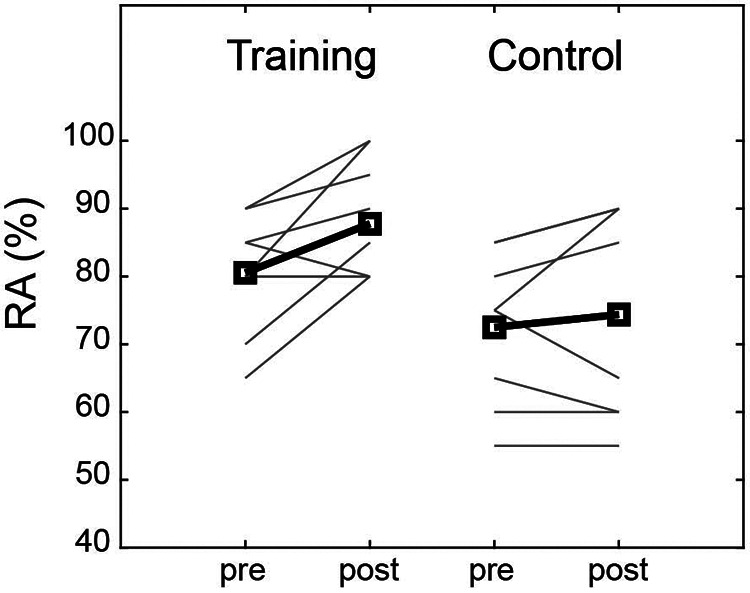
Response accuracy pre- and post- perceptual training. Group mean of response accuracy (bold lines and squares) and individual changes (solid lines) for the training and control group.

In the training group, RA was improved by an average of 9% relative to the baseline. This corresponded to an improvement of 8% compared to the control group, and the effect size was large (Table 2). Six of the training group members showed improvement, whereas the control group showed no consistent change (Figure 2).

**Table 2:** Intervention effects and group differences for RA and delta onset.

Variable	Training (*n* = 9)	Control (*n* = 8)	Cohen's *d*
RA (%)			
Intervention effect [95% CI]	109 [101 118]	101 [93 110]	
Group difference [95% CI]	108 [96 121]		0.748
Delta onset (%)			
Intervention effect [95% CI]	140 [98 200]	101 [70 148]	
Group difference [95% CI]	138 [82 232]		0.649

The intervention effects were values of geometric means, which is *Y* in Equation (1), followed by exponential transformation. The group difference was calculated using Equation (2). These values were adjusted by ANCOVA analysis.

### 3.2 Delta onset

**Figure 3:**
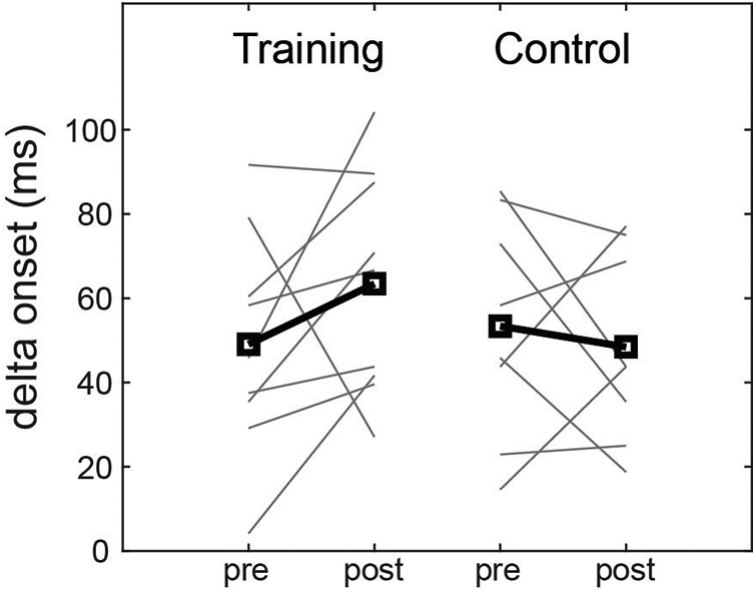
Delta onset pre- and post- perceptual training. Group mean of delta onset (bold lines and squares) and individual changes (solid lines) for the training and control group.

The training group's delta onset improved by an average of 40% relative to the baseline. This corresponded to an improvement of 38% compared to the control group, and the effect size was moderate (Table 2). Seven of the training group members showed improvement, whereas the control group showed no consistent change (Figure 3).

### 3.3 Relationships between response accuracy and delta onset

**Figure 4:**
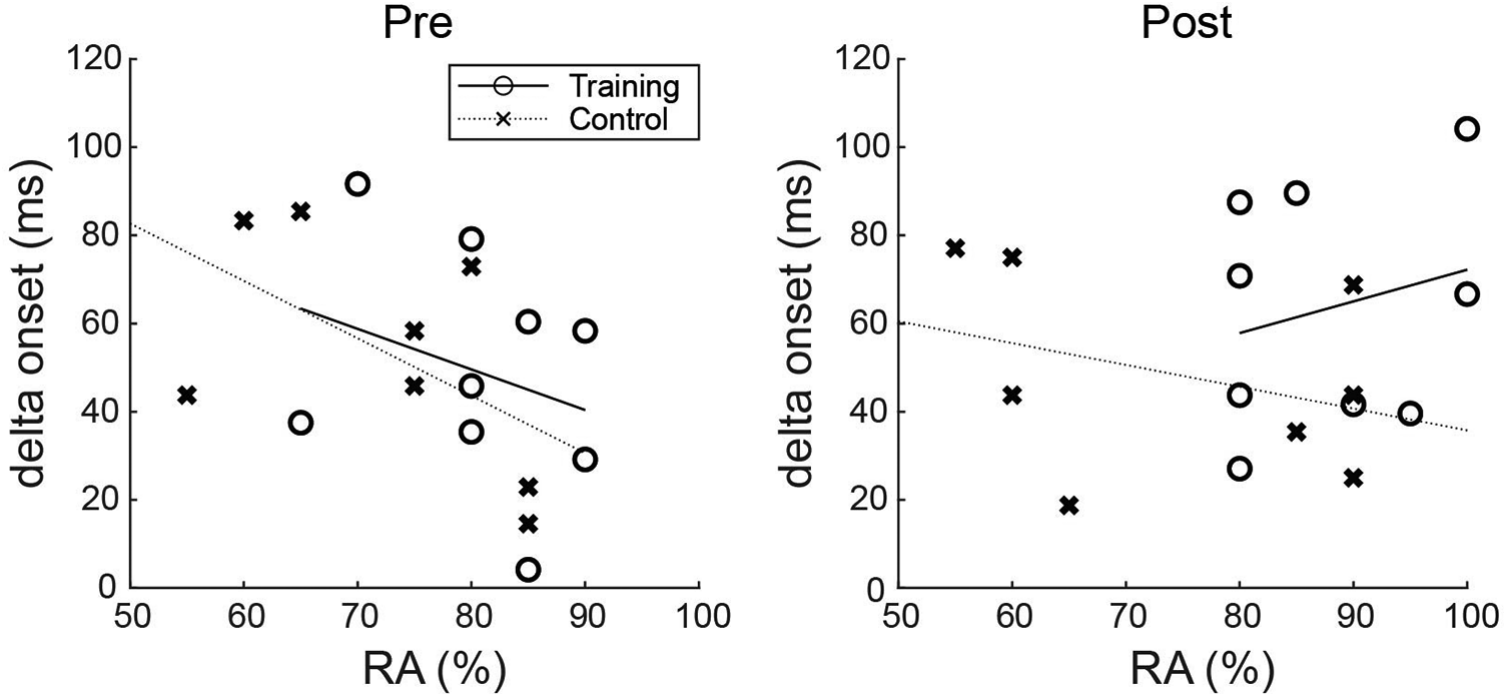
Relationships between response accuracy and delta onset pre- and post-training. The relationship between the response accuracy and the delta onset changed from negative to positive in the training group, but not in the control group.

The relationship between the perception and action test outcomes was examined (RA and delta onset, respectively; Figure 4). The results showed that the correlation coefficients of the training group changed from negative to positive values pre- and post-training (Pearson's r for the training group, pre: −0.292, post: 0.233; control group, pre: −0.557, post: −0.343). This indicated that participants with better perceptual anticipation were less adjusted for their swing onset pre-training. However, the relationship changed to one in which the participants with better perceptual anticipation were more adjusted to their swings post-training.

## 4 Discussion

This study aimed to investigate whether a VR perception-only training intervention improved swinging action (delta onset) as well as perception (RA) regarding the ability to anticipate ball speed during softball batting in the VR. The results indicated that the intervention effect of the perceptual training was higher in the training group than in the control group for both RA and delta onset.

The baseline RA for the training group was approximately 80% (Figure 2), which was higher than that of similar previous studies (50%–60%) (26, 32, 33). The pitchers in this study belonged to the same team as the participants and were familiar with them, which may explain the high RA at baseline. It is usually expected that the higher the baseline, the smaller the intervention effect; however, the participants showed an improvement of 9%. The effect size was large enough to indicate that the perceptual training effectively improved perception.

Delta onset improved by 40%. This corresponded to an increase of approximately 14.4 ms in the delay of the swing onset time for a slowball compared to a fastball (Figure 3). If a batter faced a 70 km/h slowball, she could now delay her onset by 0.28 m in distance, which meant waiting for the equivalent of three softballs in diameter and avoiding possible missed swings. Thus, perceptual training could be considered to improve swinging action.

The effects of evaluation and training of anticipatory skill without action in fastball sports have been the subject of debate (15, 16). However, the hypothesis that the two visual pathways are independently associated with perception and action, on which these claims were based, is now revisited. The hypothesis that the two visual pathways, which have many interconnections (19, 22), are processed separately seems overstated, and the assumption that one of the visual pathways is dominant depending on the attributes of the visual information presented is more plausible (20, 21).

Therefore, the current study attempted to demonstrate that batters could improve their motor skills through perception-only training if the training environment stimulated a visual system focused on action processing. To achieve this, the progressive occlusion paradigm was adopted. This study hypothesized that training solely based on pitching kinematics, without ball flight information, could lead to a fixation on form discrimination. Thus, it was important to provide a sequence of information that included both pitching kinematics and ball trajectory. The occlusion time in the training session was gradually preponed based on the training step, although ball flight information was visible for the majority of the training period (occlusion at ball release was only conducted for the last session). This approach ensured that attributes easily associated with action were accessible for most of the training period.

Mann et al. (26) argued for the importance of the coupling of perception and action with the result that cricket players predicted the ball direction better with action than without action when they predicted it based only on the opponent's kinematics. However, the coupling of perception and action was not important *per se*, but rather the use of the available visual information (i.e., opponent kinematics) for action and not for perception. Since the opponent's kinematics had both “form” and “motion” attributes, it could be processed on either the ventral or dorsal pathway (22). Some studies examining the effect of applying visual blurs (i.e., manipulation of visual attributes) to the opponent's kinematics on prediction accuracy in ball sports (17, 34) have indicated that certain levels of visual blurring increased the accuracy of verbal predictions, even just from the opponent's kinematics. This was interpreted as resulting from switching the participants' predictions from being based on clear information (fine acuity) to available and functionally meaningful information (coarse acuity). In the current study, the correlation coefficients between perception and action test outcomes changed from negative to positive values pre- and post-training, only for the training group (Figure 4). Since the opponent's kinematics was the only source of information in the perception test, the results indicate that the training enabled participants to use the information from the opponent's kinematics in their hitting action. We interpreted our results to indicate that most participants concentrated on identifying the shape of the opponent's kinematics in the perception test pre-training but changed their view of the opponent's kinematics to information that could be used for hitting the ball post-training. This may be the result of a switch in the visual processing of the opponent's kinematics from the ventral to the dorsal pathway, or it may be the result of a strengthening of the connectivity between the two pathways.

The VR system allowed participants to perceive depth in the ball trajectory and surrounding environment, likely resulting in a highly immersive experience. In a questionnaire survey of players using the same VR system as in this study, positive feedback was obtained regarding the high level of immersion, the realism of the pitcher and ball trajectory, and the perceived effectiveness compared to 2D screens (28). Such a high level of immersion provided by VR may have facilitated participants' ability to imagine hitting the ball. Indeed, one study reported that the temporal behavior of baseball batting in the HMD-VR is closer to the behavior in real situations than in the 2D display (27). However, this study did not include a transfer test; therefore, there is no direct assurance that this training will improve real-game performance. While this does not affect the discussion of whether perception-only training improves action, it should be acknowledged that the results are limited to the VR environment.

In addition, the participants in this study were highly trained players. If the participants were novices who had not been trained to hit a ball thrown by a pitcher (i.e., they had no experience in interpreting opponent kinematics for the action), they may be unsuccessful in recognizing the type of throw, regardless of the level of realism provided by VR or other means. As suggested by van der Kamp et al. (15), employing perceptual training to novices may be fruitless, as their motor execution would rely on the ventral system and not be highly automatic.

This study had several limitations. First, since it was not possible to include a placebo group, the criticism that the training group simply became familiar with the VR environment used both in the tests and training is unavoidable. However, it has been demonstrated that the VR system used in this study can be evaluated equally well with a real environment without the need for a long familiarization period (28). Therefore, we believe that the results of this study are not solely based on the familiarization with the VR system. Second, hypothesis testing was not conducted due to the small sample size. If a general hypothesis test had been conducted, the results might have been dismissed. However, we strongly believe that, in studies involving sports training, considering the meaning of the substantive effects is paramount, rather than discarding the data due to hypothesis testing. It is difficult to deny that the scientific basis for the generalization of this study's results is weak; therefore, it is necessary to accumulate evidence through a continuous study.

The results of this study do not contradict the concept that training in a more realistic environment is more effective than perceptual training. It has been reported that evaluation and training with visual stimuli and actions that are closer to those of real-world games can more accurately assess actual sports performance, and the training is more effective in such cases (5, 10, 35–37). Perception should be affected by actual actions, following the situations and time constraints in the sport or by the individual's ability to act (7, 38–40). Therefore, perceptual training is an addition to realistic training with actions. However, in sports, conducting training in an environment close to the actual game situation is rather rare, and it is common to separate the parts and train them in smaller segments (e.g., training with a baseball batting tee). In this context, it is significant that this study demonstrated the possibility of the effectiveness of perception-only training. We believe that it will contribute to the advancement of perceptual training and the use of VR in sports.

## Data Availability

The raw data supporting the conclusions of this article will be made available by the authors, without undue reservation.
